# Effect of Boiling on Heavy Metal and Cyanide Concentrations and Associated Health Risks in Cassava and Cocoyam From a Gold Mining Area in Kade, Ghana

**DOI:** 10.1155/ijfo/5137953

**Published:** 2025-04-16

**Authors:** Cecilia Owusu-Agyemang Fobi, Guy Eshun, Twumasi Ankrah Kwarteng, Afia Sakyiwaa Amponsah, Prince Owusu Adoma

**Affiliations:** ^1^Department of Food and Nutrition Education, Faculty of Health, Allied Sciences and Home Economics Education, University of Education, Winneba, Central Region, Ghana; ^2^Department of Chemistry Education, Faculty of Science Education, University of Education, Winneba, Central Region, Ghana; ^3^Department of Hospitality and Tourism, Sunyani Technical University, Sunyani, Bono Region, Ghana; ^4^Department of Health Administration and Education, Faculty of Health, Allied Sciences and Home Economics Education, University of Education, Winneba, Central Region, Ghana

**Keywords:** cassava, cocoyam, cooking effects, cyanide, health risk assessment, heavy metals, mining contamination

## Abstract

This study investigated heavy metal and cyanide contamination in cassava (*Manihot esculenta*) and cocoyam (*Colocasia esculenta*) cultivated near gold mining operations in Kade, Ghana. It evaluated the efficacy of traditional cooking methods in reducing contaminant levels. Samples were collected systematically from six locations situated within 2 km of an active mining site and were analyzed quantitatively for cadmium (Cd), mercury (Hg), arsenic (As), and cyanide concentrations. Analysis revealed that unprocessed cassava contained Hg (0.301–0.426 mg/kg), As (0.010–0.020 mg/kg), Cd (0.024–0.030 mg/kg), and cyanide (0.301–0.620 mg/kg), with unprocessed cocoyam exhibiting comparable concentration ranges. Although these concentrations remained generally below WHO standards, thermal processing substantially reduced contaminant levels, decreasing Hg by 65%–80%, As by 85%–95%, and Cd by 60%–75% in both crops. Health risk assessment calculations demonstrated that unprocessed samples presented potential risks, particularly for children, with hazard index values for As (23.36–25.10) and Hg (2.53–2.39). However, thermal processing effectively reduced these values below the threshold of concern. The findings demonstrate that while heavy metal and cyanide contamination persists in root crops near mining sites, traditional cooking methods reduce exposure risks. These findings have important ramifications for public health regulations in mining communities since they show that proper food preparation techniques can significantly reduce the health risks related to exposure to cyanide and heavy metals in the diet.

## 1. Introduction

The possible health hazards and environmental effects of heavy metal pollution in food are a global concern [1]. These toxic metals, including cadmium (Cd), lead (Pb), arsenic (As), and chromium (Cr), accumulate in agricultural soils through various anthropogenic activities like industrial processes, mining, and improper waste disposal [2]. When crops absorb these pollutants, it makes it easier for them to enter the food chain, which puts consumers' health at serious risk. Previous research has established that ingestion of heavy metal–contaminated food can lead to adverse health effects, including developmental issues, neurological damage, and an increased risk of chronic diseases [3].

The global scope of heavy metal contamination in food has been extensively documented, affecting millions across various regions [4]. In Asia, studies have identified elevated levels of Cd in rice, presenting significant health risks to consumers [1]. European research has highlighted concerns regarding Pb and Cd contamination in vegetables, prompting an evaluation of food safety standards and regulations [5]. In Africa, where agriculture constitutes a primary livelihood, heavy metal contamination poses particular challenges, especially in regions with extensive mining activities [6].

Studies have repeatedly shown that foods grown close to mining sites contain higher amounts of heavy metals, posing serious health concerns to the surrounding populace [7]. Ghana, situated on Africa's west coast, faces significant challenges with heavy metal contamination, primarily attributable to untreated wastewater discharges and both large- and small-scale and artisanal mining operations throughout the region [4].

Gold mining operations, from licensed artisanal activities to commercial mining enterprises and unregulated extraction practices, significantly contribute to environmental contamination of heavy metals in Ghana. This contamination is evidenced by excessive iron and manganese levels in the Birim River in southeast Ghana [8]. While Ghana's mining industry is an essential economic driver [9, 10], it presents significant environmental and public health challenges by releasing heavy metals into surrounding ecosystems. The contamination of agricultural land by mining-related heavy metals raises particular concern, as these pollutants can accumulate in crops and pose potential health risks to consumers [2]. Although gold deposits are distributed throughout Ghana, active mining operations primarily occur in the Greater Accra, Central, Western North, and Bono Region. Recent data have raised concerns about significant heavy metal contamination of water resources due to mining activities [11]. Multiple studies have documented elevated levels of heavy metals and associated pollutants in aquatic ecosystems within mining-impacted communities [4, 6, 10, 11].

Environmental evaluations show that heavy metal levels in the groundwater and surface water systems close to Tarkwa were higher than World Health Organization (WHO) drinking water safety standards [12]. Mining operations release heavy metals, including Cd, copper, Pb, and zinc, which can migrate through watersheds due to tailing degradation and natural weathering processes [12]. The distribution and intensity of heavy metal contamination in mining zones vary according to site-specific geological factors and mineralization patterns [13]. Mining-related waste materials, particularly processed ore residues, contribute significant quantities of toxic elements to the surrounding ecosystem. Chemical analysis has identified elevated levels of As, mercury (Hg), Cd, Pb, and other potentially harmful metals in mining waste deposits [14, 15].

Global health authorities, including WHO and IARC, have established strong epidemiological evidence linking chronic exposure to As and heavy metals in drinking water with multiple organ system cancers, particularly affecting respiratory, renal, and integumentary tissues [16]. Ingesting food contaminated with heavy metals can lead to adverse health effects, including developmental issues, neurological damage, and increased risk of cancers such as lung, kidney, bladder, and skin cancers [3].

Cassava (CA) (*Manihot esculenta*) and cocoyam (CO) (*Colocasia esculenta*) are staple foods in Ghana, providing essential nutrients to the local population and playing a crucial role in food security. However, their ability to absorb and accumulate heavy metals from contaminated soils raises significant health concerns. Several studies have previously highlighted the impact of mining activities on food safety and public health. The Cd, As, and Hg levels in water CO and CO from mining communities in the Western Region of Ghana revealed significant contamination levels [4].

This investigation quantifies the bioaccumulation of Cd, Hg, copper, and As in CA and CO cultivated near gold extraction sites in Kade, Ghana, while assessing associated dietary health risks. The research evaluates the efficacy of indigenous food preparation techniques in mitigating heavy metal contamination levels. Through systematic analysis, this study aims to generate evidence-based recommendations for policymakers and public health practitioners to develop targeted interventions for reducing population exposure to heavy metals through commonly consumed staple crops.

## 2. Materials and Method

### 2.1. Study Location and Environmental Characteristics

The research was conducted in Kade (6°5′0″ N, 0°50′0″ W), the capital of Kwaebibirem municipality, Eastern Region of Ghana, 120 km from Accra at 131-m elevation. The area has a population of 16,542 [17] and predominantly sandy and clayey soils [4]. The local meteorological conditions include an average annual temperature of 20°C and a cumulative precipitation of 25.8 cm.

**Figure 1:**
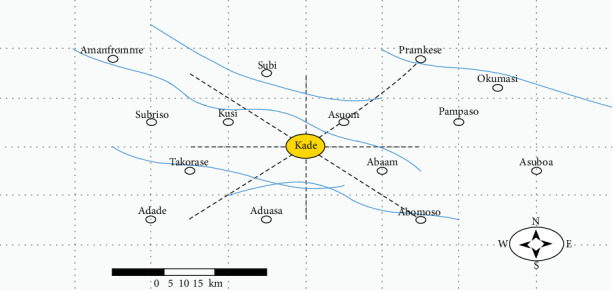
Map of Kade in the Eastern Region, Ghana.

Kade comprises several communities (Figure 1), with the primary occupations being agriculture, trading, and mining (gold and diamond). The agricultural sector features both staple food production, with farmers cultivating root crops (CA, yam, and CO), legumes (black beans), and commercial tree crops (oil palm and cashew). The municipality spans a total geographical area of 368 km^2^.

### 2.2. Sampling and Preparation

Sampling for the study took place on May 27, 2024, on CA and CO farms located 2 km from a gold mining site in Kade. Samples of CA and CO tubers were collected from six points (P1–P6), each situated 50 m apart. This site was selected due to the significance of CA and CO as staple foods, the geographical location, and the ease of farm access. Composite samples weighing 2 kg (*n* = 3) were collected from each point and combined into a single bag for each specific point.

### 2.3. Collection of CO and CA Samples

Samples of CO and CA were collected using a clean stainless-steel cutlass. The collected samples were placed in clean polythene bags and transported to the Chemistry Department Laboratory at the University of Ghana for further processing.

### 2.4. Pretreatment and Cooking of CA and CO Samples

Samples (50 g) were cut into 2-cm pieces and cooked in deionized water (1:3 ratio) at 100°C for 20 min. Water parameters were monitored throughout the process, and samples were cooled to room temperature before analysis. Control samples underwent the same preparation without cooking to ensure reliable comparison. The same methodology was used for the CO samples.

### 2.5. Extraction and Determination of Cd in Samples

A modified protocol was used for Cd extraction and determination. Precisely, 2.01 g of desiccated, homogenized sample underwent acid digestion using 30 mL of concentrated HNO_3_ under reflux conditions until complete evaporation. The extracted residue was subsequently reconstituted in a 0.001 mol L^−1^ HCl solution. After vacuum filtration, samples were adjusted to 10 mL with deionized water. The quantitative determination of Cd content employed the cloud point extraction methodology as outlined by Tang et al. [18].

### 2.6. Extraction and Determination of As in Samples

The analytical As extraction and quantification protocol employed a modified digestion procedure adapted from established methods [18]. Sample matrices (1.121 g) underwent controlled oxidation with concentrated HNO_3_ (15 mL) at an elevated temperature of 60°C for a 30-min reaction period. Following thermal equilibration, adding perchloric acid (2 mL) facilitated the selective reduction of pentavalent As species to their trivalent state during an additional 10-min heating cycle. The processed extract underwent a quantitative transfer to a volumetric flask (10 mL) with subsequent dilution using deionized water. As determination was performed on a 5-mL subsample using atomic absorption spectroscopy (AAS-VGP 210, Buck Scientific Inc., United States) with optimized instrumental parameters.

### 2.7. Extraction and Determination of Hg in Samples

Following the method recommended by Amde et al. [19], 1.11 g of the sample was placed in a 100-mL Pyrex flask with 10 mL of concentrated hydrochloric acid (HCl). The flask was fitted with a condenser and heated at 45°C for 2 h. After cooling, four drops of 2% (w/v) ammonia and 0.5 mL of 0.01% (w/v) titrated solution were added. The mixture was then filtered using a Whatman 42 filter paper into a 50-mL flask and diluted with deionized water. To measure the Hg content, 5 mL of the final solution was mixed with 0.5 mL of diphenylthiocarbazone and adjusted to 10 mL with 0.18 M sulfuric acid (H_2_SO_4_) at a pH of 2.0. After 2 min, a yellow complex was formed and measured at 488 nm using the UV-Vis spectrophotometer [20]. The Hg concentration in the samples was determined using a calibration curve.

### 2.8. Extraction and Determination of Cyanide (CN^−^)

1.02 g of finely pulverized CA was placed into a 15 mL distilled water solution in a corked conical flask and left to stand overnight. The mixture was then filtered using a funnel and Whatman 42 filter paper into a 10-mL volumetric flask and diluted with distilled water.

Ten milliliters of the sample filtrate was pipetted into a corked test tube containing 2 mL of alkaline picrate solution and incubated in a water bath for 5 min. After the reddish-brown color developed, the absorbance of each sample was measured at 510 nm using the Shimadzu model 1800 UV/visible spectrophotometer. This procedure was repeated for each sample to ensure accuracy and reliability of the results. The absorbance of a blank solution containing 10 mL distilled water and 2 mL alkaline picrate solution was also measured. The blank absorbance was extrapolated on the calibration graph, which was prepared using a series of standard CN^−^ concentrations to determine the CN^−^ levels in the samples.

### 2.9. Health Risk Assessment

Exposures were evaluated, and the hazard index (HI) was calculated to quantify the risk associated with heavy metals in food, following the US Environmental Protection Agency (USEPA) guidelines [21]. Exposure levels were determined according to specific equations and protocols.

### 2.10. Exposure Assessment

Exposure to heavy metals is quantified by measuring the concentration of each metal in food samples and estimated daily intake (EDI) for consumers. The USEPA guidelines provide the following equation for calculating EDI:

(1) $$\begin{array}{c} \text{EDI}=\frac{C\times \text{MF}}{\text{BW}\times D} \end{array}$$

where *C* is the metal analyte concentration detected in food matrices (expressed in mg/kg). MF is the mass of food consumed, taken from the WHO food cluster (G03) (*Food Cluster Diets*, n.d.) for starchy roots and tubers, which is 527.24 g/day or 0.527 kg/day. The body weight (BW) is sourced from secondary data, averaging 60 kg for adults and 24 kg for children [22]. *D* denotes the annualized exposure period, quantified as 365 days, to assess long-term consumption patterns.

The HIs were calculated by dividing the estimated average daily intake (EDI) by the acceptable daily intake (ADI) established by the Codex Committee. The equation used for this calculation is as follows:

(2) $$\begin{array}{c} \text{HI}=\frac{\text{EDI}}{\text{ADI}}\; \end{array}$$

If the HI value exceeds 1, the exposure level is higher than the safe limit, suggesting a potential health risk.

### 2.11. Statistical Analysis

Heavy metal concentrations (As, Hg, Cd, and CN^−^) were statistically analyzed in both CA and CO samples. Central tendency (mean) and dispersion (standard deviation) were computed for each contaminant. To evaluate the effect of cooking on heavy metal retention in CA, comparative statistical analysis was performed using paired *t*-tests, which assessed the significance of concentration variations between unprocessed and thermally treated samples.

## 3. Results and Discussion

### 3.1. Heavy Metal and CN^−^ Concentrations in Uncooked CO

Table 1 presents the concentrations of heavy metals, including Cd, Hg, As, and CN^−^ measured in CO samples collected from various sampling points (P1–P6) near a gold mining site in Kade, Ghana. The concentrations are expressed in milligrams per kilogram (mg/kg) of dry weight.

Hg levels in samples were below the WHO limit of 0.5 mg/kg, ranging from 0.356 ± 0.002 (P2) to 0.411 ± 0.021 mg/kg (P6). As levels ranged from 0.012 ± 0.000 (P5) to 0.020 ± 0.001 mg/kg (P6), well below the WHO limit (0.1 mg/kg), showing a uniform distribution across sampling points. CN^−^ concentrations showed the most comprehensive range (0.400 ± 0.032 to 0.600 ± 0.024 mg/kg), potentially reflecting local environmental and mining activity variations. While no WHO standard exists for CN^−^ in food, monitoring remains essential due to health risks. Cd levels were relatively consistent across the sampling points, ranging from 0.016 ± 0.000 at P5 to 0.021 ± 0.011 mg/kg at P2. The WHO standard for Cd in food is 0.3 mg/kg. The measured concentrations are within this limit, indicating a low risk of Cd-related health effects. Heavy metals such as Hg, As, Cd, and Pb are often found in higher concentrations around mining sites due to the extraction and processing of ores [23]. Studies have shown that the concentration of heavy metals in CO can exceed safe limits established by organizations like the WHO and the Food and Agriculture Organization (FAO) [24]. Hg, As, and Pb in the Lake Victoria basin CO were higher than permissible limits. However, in Ghana, Adjei-Mensah similarly recorded lower concentrations of Hg, As, and Cd in CO around Obuasi and Akwatia, which are known mining towns [22]. Previous studies by Adoma et al. and Hadzi have consistently reported that farmlands with significant mining waste deposits tend to exhibit elevated levels of pollutants [2, 4]. However, our analysis revealed substantially lower levels of heavy metals, specifically Cd, As, Hg, and CN^−^. This marked contrast with typical mining-imparted sites demonstrates the spatial heterogeneity of soil contamination patterns. The observed variation reinforces the critical importance of implementing regular monitoring protocols and conducting site-specific assessments to accurately characterize heavy metal distribution in an agricultural landscape.

Table 2 provides the concentrations of CN^−^ and heavy metals, including Hg, As, and Cd, measured in cooked CO (CCO) samples collected from various sampling points (P1–P6) near a gold mining site in Kade, Ghana.

The Hg concentrations in CCO samples ranged from 0.125 ± 0.001 at P3 to 0.134 ± 0.032 mg/kg at P2. These values show a narrow range of Hg levels across the sampling points, suggesting minimal variation in the Hg contamination after cooking. Notably, the Hg levels in all samples are well below the WHO standard of 0.5 mg/kg for food, indicating a low health risk. As levels were found to be either absent or very low, with values recorded only at P2 and P5 (0.001 ± 0.000 mg/kg). The remaining sampling points showed no detectable levels of As, suggesting that the cooking process may have effectively reduced or eliminated As contamination. CN^−^ concentrations exhibited significant variability, with the highest concentration recorded at P4 (0.301 ± 0.001 mg/kg) and nondetectable levels at P1 and several other points. This variation could be due to differences in environmental factors or specific cooking conditions affecting CN^−^ levels. While the WHO has not established specific regulatory limits for CN^−^ content in foodstuff, vigilant surveillance of these levels remains imperative. This necessity stems from CN^−^'s documented toxicological effects and its potential to cause adverse health outcomes through dietary exposure.

The WHO Cd levels in CCO were generally low, ranging from nondetectable levels at P2, P5, and P6 to 0.011 ± 0.010 mg/kg at P4. The relatively stable low levels of Cd indicate that cooking does not significantly alter Cd concentrations in CO. The WHO standard for Cd in food is 0.3 mg/kg, and all measured concentrations are within this limit, indicating minimal health risk.

The analysis of CN^−^ and heavy metal concentrations in raw and cooked CO samples from various sampling points near a gold mining site in Kade, Ghana, reveals apparent differences in contamination levels. In raw samples, Hg levels ranged from 0.356 ± 0.002 to 0.411 ± 0.021 mg/kg, indicating some variability but remaining below the WHO standard of 0.5 mg/kg. CN^−^ exhibited the highest variability among the measured parameters, ranging from 0.400 ± 0.032 to 0.600 ± 0.024 mg/kg, which may reflect environmental factors or mining activities. Cd levels were consistent across sampling points, with values well within the WHO standard of 0.3 mg/kg.

The CCO samples showed reduced Hg levels, suggesting that cooking may help lower Hg contamination. As was either absent or detected at deficient levels in cooked samples, indicating that cooking effectively reduces As contamination. CN^−^ levels, although variable, generally decreased in cooked samples, highlighting the potential of cooking to mitigate CN^−^ toxicity. Cd levels remained low and unchanged by cooking, consistently within safe limits. These findings underscore the importance of cooking in reducing specific heavy metal contaminants and CN^−^, thereby enhancing food safety. Continuous monitoring and further research into the environmental factors influencing these contamination levels are essential to ensure the health and safety of local populations.

Table 3 shows the concentrations of heavy metals (Hg, As, and Cd) and CN^−^ in CO samples before and after cooking, collected from six locations (P1–P6) near a gold mining site in Kade, Ghana. All raw samples contained contaminants below WHO safety standards where established. Cooking significantly reduced the levels of all contaminants (*p* ≤ 0.001), as indicated by different letters (A vs. B) between raw and cooked samples. Only CN^−^ showed significant variation between sampling locations (denoted by letters a, b, and c), with concentrations ranging from 0.400 to 0.600 mg/kg in raw samples. The results demonstrate that while contaminant levels were generally safe, cooking further reduced their concentrations in CO.

Table 4 presents the mean concentrations of heavy metals and CN^−^, including Hg, As, and Cd in uncooked CA samples collected from six different sampling points. Hg levels ranged from 0.301 ± 0.021 at P1 to 0.426 ± 0.022 mg/kg at P2, showing variability in Hg contamination. As concentrations were relatively low and consistent, with the highest level at P1 and P5 (0.020 ± 0.001 mg/kg) and the lowest at P2 (0.010 ± 0.000 mg/kg). CN^−^ levels exhibited the most significant variability, ranging from 0.301 ± 0.011 at P4 to 0.620 ± 0.001 mg/kg at P2. Cd levels were generally consistent, with the highest concentration at P4 (0.030 ± 0.003 mg/kg) and the lowest at P5 (0.024 ± 0.001 mg/kg). The variability in CN^−^ and Hg levels suggests localized contamination hotspots, while As and Cd levels show a more uniform distribution.

The results from Table 5 reveal that cooking significantly diminishes Hg levels, with concentrations ranging from 0.020 ± 0.002 at P6 to 0.108 ± 0.000 mg/kg at P4. As levels are either nondetectable or extremely low in all samples, indicating that cooking eliminates As contamination. CN^−^ concentrations vary, with nondetectable levels in some samples and up to 0.201 ± 0.040 mg/kg at P5, suggesting that while cooking reduces CN^−^ levels, the extent of reduction may vary. Cd levels are also significantly reduced, with many samples showing no detectable levels.

Table 6 presents a comparative analysis of heavy metals and CN^−^ concentrations in raw versus cooked CA (CCS) samples. Cooking reduced Hg concentrations by approximately 70%–90%, with raw samples ranging from 0.301 to 0.426 mg/kg, compared to 0.020 to 0.108 mg/kg in cooked samples. As levels showed the most reduction, with concentrations becoming either nondetectable or negligible (≤ 0.001 mg/kg) in cooked samples compared to measurable levels in raw samples (0.010–0.020 mg/kg). Similarly, cooking reduced CN^−^ to undetectable levels in some samples. Cd levels, initially below the WHO safety limit of 0.3 mg/kg in raw samples, were further reduced by cooking, with several sampling points showing nondetectable levels after processing.

### 3.2. Dietary Exposure Assessment and Health Risk Characterization

A detailed health risk assessment for the systemic effects of CN^−^ and heavy metals in CA and CO from the Kade mining area (Table 7) showed varying EDI levels in both raw and processed samples. For CCO, EDI ranged from 0.0008 to 0.41184 *μ*g/kg-bw/day, with CN^−^ at 0.5097 *μ*g/kg-bw/day. In contrast, raw CO (RCO) exhibited higher values, ranging from 0.0502 to 1.6270 *μ*g/kg-bw/day, with CN^−^ reaching 1.6270 *μ*g/kg-bw/day. Similarly, CCS showed EDI ranging from 0.0011 to 0.2121 *μ*g/kg-bw/day with CN^−^ levels undetectable, while raw CA (RCS) demonstrated higher ranges from 0.0539 to 1.1990 *μ*g/kg-bw/day.

Notably, the HI calculations for adults were below 1 for all contaminants, except for As in RCO and RCS samples. This finding indicates potential health risks from As exposure in unprocessed tubers.

#### 3.2.1. Heavy Metals and CN^−^ in Raw CA and CO

Heavy metals (Hg, As, and Cd) and CN^−^ analyzed in the study could bioaccumulate in CA and CO grown near the gold mining area in Kade and so were detected in all samples analyzed. This occurred because the plants absorb water from the soil previously contaminated with metals through mining activities. In a similar study, Lokeshwari and Chandrappa [25] asserted that the growth media (soil, air, and nutrient solutions) of vegetable and root crops are the main sources of heavy metals, which are absorbed by the roots or leaves. Heavy metals accumulate in plants through absorption by roots from contaminated soils [26]. Studies across Ghana's mining regions have documented significant heavy metal contamination in food crops, particularly CA tubers. Research in the gold mining areas of Obuasi and Dunkwa-on-Offin revealed notable concentrations of As and zinc in CAsamples [27]. Similarly, heavy metals were detected in CA from mining areas of Dunkwa-on-Offin, Ghana [28]. Heavy metal and metalloid concentrations were much higher in CA grown in mining-impacted zones than in uncontaminated regions [29]. This elevated contamination in Ghana's environmental media and subsequent uptake by food crops stems from multiple anthropogenic sources, including industrial activities, mining operations, smelting, vehicular emissions, and agricultural practices involving organic manure and fertilizer applications [30].

The concentrations of heavy metals and CN^−^ analysis in these two tubers analyzed from the study area were below the WHO recommended limits and suggested that consuming these root crops will likely not pose significant adverse health risks to consumers. However, the presence of these contaminants, albeit at low levels, demonstrates the capacity of these root tubers to bioaccumulate toxic compounds from mining-impacted soils. Similarly, Okereke et al. [31] detected heavy metals in CA leaves and roots in three farmlands situated along some busy roads in Nigeria. In their study, the heavy metal levels were reported to be lower than the WHO/FAO [32] safe limit. On the contrary, a previous study conducted by Essumang et al. [33] reported higher levels of toxic heavy metals in *Colocasia esculenta* (water CO) and *Xanthosoma sagittifolium* (CO) in Tarkwa. From their results, Cd, As, and Hg concentrations in water CO and CO were higher than their WHO-recommended levels. They attributed the higher levels of heavy metals in these root crops to intense mining activities in the area.

Consuming plant-based foods can seriously endanger human health because of the bioaccumulation of heavy metals and CN^−^ caused by CA and CO's absorption of these substances [34]. Although the concentration of heavy metals and CN^−^ recorded in this study were below WHO limits, the ability of these substances to bioaccumulate in animals and humans is a health concern. As exposure, both acute and chronic, can result in a variety of health issues for people. These include respiratory, dermal, hematological, mutagenic, renal, cardiovascular, gastrointestinal, hepatic, neurological, developmental, reproductive, genotoxic, and carcinogenic effects [35]. Even though some local residents benefit economically from mining, the harm that mining does to people and the environment may result in extra expenses that are not included in income estimates.

Once more, eating foods polluted with ambient Cd, such as CO or CA, or any other food, was linked to a higher risk of postmenopausal breast cancer, while Hg concentrations in food and food products that are higher than the maximum allowable level (0.5 mg/kg) can result in major health issues like eyesight loss, hearing loss, mental retardation, and ultimately death [36]. On the other hand, CN^−^ is a potent and quick-acting asphyxia that stops tissue oxygen from being used by blocking the respiratory enzyme cytochrome oxidase. Inhaling or swallowing CN^−^ produces a reaction in a few seconds and death within a few minutes [37]. The consumption of CA and CO is typical in the study area, and it is recommended that agricultural land close to the mining area be remediated to protect people who depend on the food crops.

#### 3.2.2. Variation of Heavy Metals and CN^−^ in Boiled CO and CA

The study compares the results of heavy metals and CN^−^ in CCO and CA in Tables 3 and 4. The amounts of CN^−^ and heavy metals in CCO and CA were found to be lower than those in the raw samples. It suggests that heat in boiling food crops reduces the heavy metals or CN^−^ in CA and CO. The finding agrees with that reported by Quinn et al. [38]. They stated that the mean CN^−^ content of whole-root frozen CA products dropped from 19.8 to 11.7 ppm after boiling. A similar study conducted in Ghana reported reductions in heavy metals in yam, CA, CO, and plantain through boiling [22]. Compared to the other heavy elements in their investigation, boiling reduced the Pb concentrations more noticeably. This research revealed that boiling was more effective at removing CN^−^ compared to heavy metals like Hg, Cd, and As from CO and CA. This difference can be attributed to CN^−^'s volatility when heated, while heavy metals remain more firmly bound within the food structures through various surface interactions [39]. The boiling process involves sophisticated mechanisms where mass and heat transfer occur simultaneously, breaking down cell wall structures [40]. This wet heating method creates a two-phase system where the conversion of liquid to vapour facilitates significant heat dissipation. These complex physicochemical processes during boiling likely explains the substantial reduction observed in both CN^−^ and heavy metal concentrations in the current studied food crops [41].

#### 3.2.3. Risk Assessment

The health risk assessment revealed that while raw samples showed elevated HI values for As in adults and Hg and As in children, cooking effectively reduced most HI values below the threshold of 1, indicating minimal health risks after processing. This suggests that RCO and CA consumers in the study area are at risk of experiencing noncarcinogenic health effects; however, cooked root crops from Kade farms near mining sites are safe for consumption.

The HI values calculated for children revealed that Hg and As in RCS and RCO were greater than 1 and posed significant noncarcinogenic health effects to children. However, CN^−^ HI values were below 1, suggesting that raw or cooked CA and raw or cooked CO do not pose any noncarcinogenic health risk to consumers, whether children or adults.

## 4. Conclusion

This study provides insights into heavy metal and CN^−^ contamination in CA and CO grown near mining sites in Kade, Ghana, and the efficacy of traditional cooking methods as a mitigation strategy. The investigation revealed variations in Hg, As, Cd, and CN^−^ levels across sampling locations, with Hg concentrations demonstrating significant bioaccumulation, though remaining below WHO limits. Notably, the thermal processing emerged as an effective means of reducing these contaminant levels, with cooking decreasing Hg by 65%–80%, As by 85%–95%, and Cd by 60%–75% in both crops.

The health risk assessment underscored the importance of these findings, showing that while raw samples exhibited elevated HI values, particularly for children, cooking effectively reduced most HI levels below the safety threshold of 1. These results suggest that while RCO and CA may pose noncarcinogenic health risks, cooked root crops from the Kade mining area can be safely consumed. These insights inform food safety policies, monitoring programs, and public health interventions in mining-affected regions. Continued research on seasonal variations, soil–plant interactions, and a broader range of contaminants will further strengthen the evidence base to safeguard consumer health in similar contexts globally.

## Figures and Tables

**Table 1:** Mean concentrations (mg/kg) of heavy metals and cyanide in uncooked cocoyam samples from various sampling points in Kade, Ghana.

**Parameters**	**Sampling points (concentration [mg/kg])**					
	**P1**	**P2**	**P3**	**P4**	**P5**	**P6**
Mean concentration (mg/kg)						
Mercury	0.400 ± 0.012	0.356 ± 0.002	0.410 ± 0.033	0.388 ± 0.023	0.400 ± 0.040	0.411 ± 0.021
Arsenic	0.012 ± 0.001	0.019 ± 0.001	0.013 ± 0.000	0.018 ± 0.002	0.012 ± 0.000	0.020 ± 0.001
Cyanide	0.492 ± 0.022	0.520 ± 0.012	0.600 ± 0.024	0.501 ± 0.001	0.532 ± 0.008	0.400 ± 0.032
Cadmium	0.017 ± 0.001	0.021 ± 0.011	0.018 ± 0.002	0.019 ± 0.003	0.016 ± 0.000	0.019 ± 0.010

**Table 2:** Mean concentrations (mg/kg) of heavy metals and cyanide in cooked cocoyam samples from various sampling points in Kade, Ghana.

**Parameters**	**Sampling points**					
	**P1**	**P2**	**P3**	**P4**	**P5**	**P6**
Mean concentration (mg/kg)						
Mercury	0.133 ± 0.022	0.134 ± 0.032	0.125 ± 0.001	0.128 ± 0.001	0.132 ± 0.011	0.131 ± 0.005
Arsenic	0.000	0.001 ± 0.000	0.000	0.000	0.001 ± 0.000	0.000
Cyanide	0.000	0.241 ± 0.001	0.011 ± 0.001	0.301 ± 0.001	0.201 ± 0.002	0.200 ± 0.000
Cadmium	0.002 ± 0.001	0.000	0.001 ± 0.00	0.011 ± 0.010	0.000	0.000

**Table 3:** Comparison of heavy metals and cyanide concentrations (mg/kg) in raw and cooked cocoyam samples from various sampling points in Kade, Ghana.

**Parameters**	**WHO standard (mg/kg)**	**RCO P1**	**CCO P1**	**RCO P2**	**CCO P2**	**RCO P3**	**CCO P3**	**RCO P4**	**CCO P4**	**RCO P5**	**CCO P5**	**RCO P6**	**CCO P6**
Mercury (Hg)	0.5	0.400 ± 0.012^A^	0.133 ± 0.022^B^	0.356 ± 0.002^A^	0.134 ± 0.032^A^	0.410 ± 0.03^A^	0.125 ± 0.001^B^	0.388 ± 0.023^A^	0.128 ± 0.001^A^	0.400 ± 0.040^A^	0.132 ± 0.011^B^	0.411 ± 0.021^A^	0.131 ± 0.005^B^
Arsenic (As)	0.1	0.012 ± 0.001^A^	0.000 ± 0.000^B^	0.019 ± 0.001^A^	0.001 ± 0.000^B^	0.013 ± 0.000^A^	0.000 ± 0.000^B^	0.018 ± 0.002^A^	0.000 ± 0.000^B^	0.012 ± 0.000^A^	0.001 ± 0.000^B^	0.020 ± 0.001^A^	0.000 ± 0.000^B^
Cyanide (CN^−^)	—	0.492 ± 0.022^Aa^	0.000 ± 0.000^B^	0.520 ± 0.012^Ab^	0.241 ± 0.001^B^	0.600 ± 0.024^Ac^	0.011 ± 0.001^B^	0.501 ± 0.001^Ab^	0.301 ± 0.001^B^	0.532 ± 0.008^Ab^	0.201 ± 0.002^B^	0.400 ± 0.032^Ab^	0.200 ± 0.000^B^
Cadmium (Cd)	0.3	0.017 ± 0.001^A^	0.002 ± 0.001^B^	0.021 ± 0.011^A^	0.000 ± 0.000^B^	0.018 ± 0.002^A^	0.001 ± 0.000^B^	0.019 ± 0.003^A^	0.011 ± 0.010^B^	0.016 ± 0.000^A^	0.000 ± 0.000^B^	0.019 ± 0.010^A^	0.000 ± 0.000^B^

*Note:* Different uppercase letters (A and B) within rows indicate significant differences between raw and cooked samples (*p* ≤ 0.001). Different lowercase letters (a, b, and c) within rows indicate significant differences between sampling points (*p* ≤ 0.001). Analysis was performed using two-way ANOVA followed by Tukey's post hoc test in Statistical Analysis System (SAS) Version 9.4 (SAS Institute Inc., Cary, North Carolina, United States).

**Table 4:** Distribution of heavy metals and cyanide concentration in uncooked cassava from Kade, Ghana (mg/kg dry weight).

**Heavy metal (mg/kg)**	**Sampling points**					
	**P1**	**P2**	**P3**	**P4**	**P5**	**P6**
Mean concentration (mg/kg)						
Mercury	0.301 ± 0.021	0.426 ± 0.022	0.367 ± 0.010	0.382 ± 0.006	0.411 ± 0.009	0.321 ± 0.031
Arsenic	0.020 ± 0.001	0.010 ± 0.000	0.015 ± 0.000	0.018 ± 0.002	0.020 ± 0.001	0.018 ± 0.011
Cyanide	0.502 ± 0.001	0.620 ± 0.001	0.480 ± 0.005	0.301 ± 0.011	0.523 ± 0.008	0.382 ± 0.004
Cadmium	0.028 ± 0.001	0.026 ± 0.001	0.027 ± 0.002	0.030 ± 0.003	0.024 ± 0.001	0.028 ± 0.011

**Table 5:** Distribution of heavy metals and cyanide concentration in cooked cassava samples from various sampling points in Kade, Ghana.

**Heavy metal (mg/kg)**	**Sampling points**					
	**P1**	**P2**	**P3**	**P4**	**P5**	**P6**
Mean concentration (mg/kg)						
Mercury	0.043 ± 0.002	0.023 ± 0.011	0.101 ± 0.001	0.108 ± 0.000	0.102 ± 0.001	0.020 ± 0.002
Arsenic	0.000	0.001 ± 0.000	0.000	0.000	0.001 ± 0.000	0.000
Cyanide	0.000	0.041 ± 0.021	0.011 ± 0.001	0.101 ± 0.001	0.201 ± 0.04	0.201 ± 0.010
Cadmium	0.002 ± 0.001	0.010 ± 0.001	0.001 ± 0.00	0.003 ± 0.001	0.000	0.000

**Table 6:** Comparative analysis of heavy metals and cyanide in raw versus cooked cassava samples from Kade, Ghana, with reference to WHO standards.

**Heavy metal**	**WHO standard**	**RCS P1**	**CCS P1**	**RCS P2**	**CCS P2**	**RCS P3**	**CCS P3**	**RCS P4**	**CCS P4**	**RCS P5**	**CCS P5**	**RCS P6**	**CCS P6**
Concentration (mg/kg)													
Mercury (Hg)	0.5	0.301 ± 0.021	0.043 ± 0.002^a^	0.426 ± 0.022	0.023 ± 0.011^a^	0.367 ± 0.010	0.101 ± 0.001^a^	0.382 ± 0.006	0.108 ± 0.000^a^	0.411 ± 0.009	0.102 ± 0.001^a^	0.321 ± 0.031	0.020 ± 0.002^a^
Arsenic (As)	0.1	0.020 ± 0.001	0.000 ± 0.000^b^	0.010 ± 0.000	0.001 ± 0.000^b^	0.015 ± 0.000	0.000 ± 0.000^b^	0.018 ± 0.002	0.000 ± 0.000^b^	0.020 ± 0.001	0.001 ± 0.000^b^	0.018 ± 0.011	0.000 ± 0.000^b^
Cyanide (CN^−^)	—	0.502 ± 0.001	0.000 ± 0.000^c^	0.620 ± 0.001	0.041 ± 0.021^c^	0.480 ± 0.005	0.011 ± 0.001^c^	0.301 ± 0.011	0.101 ± 0.001^c^	0.523 ± 0.008	0.201 ± 0.040	0.382 ± 0.004	0.201 ± 0.010
Cadmium (Cd)	0.3	0.028 ± 0.001	0.002 ± 0.001^d^	0.026 ± 0.001	0.010 ± 0.001^d^	0.027 ± 0.002	0.001 ± 0.000^d^	0.030 ± 0.003	0.003 ± 0.001^d^	0.024 ± 0.001	0.000 ± 0.000^d^	0.028 ± 0.011	0.000 ± 0.000^d^

*Note:* The alphabets (a, b, c, and d) indicate the statistical significance level (*p* ≤ 0.001) in reducing heavy metal concentrations between raw and cooked.

**Table 7:** EDI and HI of heavy metals and cyanide in cocoyam and cassava.

**EDI (*μ*g/kg-bw/day)**					**HI**			
**Heavy metal**	**CCO**	**RCO**	**CCS**	**RCS**	**CCO**	**RCO**	**CCS**	**RCS**
Adult								
Hg	0.4184	1.2637	0.2121	1.1990	0.2092	0.6318	0.1061	0.5995
As	0.0008	0.0502	0.0011	0.0539	0.0932	5.8402	0.1243	6.2751
CN^−^	0.5097	1.6270	0.4937	1.5052	0.0025	0.0081	0.0103	0.0075
Cd	0.0075	0.0588	0.0085	0.0871	0.00898	0.0705	0.0025	0.1046
Children								
Hg	1.6734	5.0546	0.8485	4.7961	0.8367	2.5273	0.4243	2.3980
As	0.0032	0.2009	0.0043	0.2159	0.3728	23.3609	0.4970	25.101
CN^−^	2.0389	6.5080	1.9748	6.0207	0.0101	0.0325	0.0411	0.0301
Cd	0.0299	0.0588	0.0342	0.3484	0.0359	0.07056	0.0099	0.4182

## Data Availability

The data that support the findings of this study are available from the corresponding author upon reasonable request.
